# Potential Mechanisms for COVID-19 Induced Anosmia and Dysgeusia

**DOI:** 10.3389/fphys.2020.01039

**Published:** 2020-09-16

**Authors:** Adrien A. Eshraghi, Mehdi Mirsaeidi, Camron Davies, Fred F. Telischi, Nirupa Chaudhari, Rahul Mittal

**Affiliations:** ^1^Department of Otolaryngology, University of Miami Miller School of Medicine, Miami, FL, United States; ^2^Department of Biomedical Engineering, University of Miami, Coral Gables, FL, United States; ^3^Department of Neurological Surgery, University of Miami Miller School of Medicine, Miami, FL, United States; ^4^Division of Pulmonary and Critical Care, University of Miami, Miami, FL, United States; ^5^Section of Pulmonary, Miami VA Healthcare System, Miami, FL, United States; ^6^Department of Physiology and Biophysics, University of Miami Miller School of Medicine, Miami, FL, United States

**Keywords:** COVID-19, anosmia, dysgeusia, cytokines, neuroinflammation, molecular mechanisms

COVID-19 is an emerging pandemic infecting more than 11 million people and leading to more than 544,722 deaths worldwide at the time of writing this report. Despite the widespread and highly contagious nature of COVID-19, the current diagnostic tests used to identify the infected patients lack sensitivity. A recent study demonstrated that sensitivity of COVID-19 tests can be as low as 59% by RT-PCR (Ai et al., 2020). Moreover, in patients with negative RT-PCRs, 75% had positive chest CT scans (Ai et al., 2020). The high rates of discordant test results and potential for false negatives in COVID-19 sometimes make it difficult for healthcare providers to identify individuals appropriately for treatment and quarantine.

Given the uncertainty in diagnostic testing, special attention to additional presenting signs of COVID-19 may be beneficial. While fever, cough, and dyspnea are common symptoms, loss of smell (anosmia) and taste (dysgeusia) are increasingly reported in asymptomatic individuals that later test positive for COVID-19. Recent studies suggested that up to 85.6% of affected patients experience anosmia, and 88.0% of affected patients experience dysgeusia. Remarkably, in at least 11.8% of cases, anosmia and hyposmia are the first presenting signs (Boscolo-Rizzo et al., 2020; Gautier and Ravussin, 2020; Lechien et al., 2020; Lee et al., 2020; Paderno et al., 2020). Some of recent case studies have reported the loss of smell or taste as the only symptom of COVID-19 (Hjelmesæth and Skaare, 2020) Thus, anosmia and dysgeusia are common but currently under-recognized early symptoms of COVID-19, often remaining inconspicuous as they may coincide with more distracting symptoms such as fatigue or cough. Additionally, in many areas, these patients do not meet the criteria for testing or self-isolation, simultaneously constituting a danger to caregivers including physicians and an opportunity to help reduce the spread of COVID-19 through early identification. Therefore, anosmia and dysgeusia may well-serve as early warning signs to identify otherwise asymptomatic individuals that could potentially pass undetected through the healthcare system without triggering COVID-19 protocols. This may be particularly useful in prioritizing patients for molecular testing or evaluating the early clinical progression or in environments where testing is limited, such as underdeveloped or rural areas. In these environments, those presenting with olfactory or gustatory dysfunction could be candidates for increased social distancing or mask-wearing until testing is available.

However, it is essential to note that the identification of anosmia and dysgeusia will not replace high-quality molecular testing in the diagnosis of COVID-19 that can even detect low copies of the virus in the testing material. Furthermore, anosmia and dysgeusia are also seen in upper respiratory infections (particularly viral infections), allergic rhinitis, chronic rhinosinusitis, and nasal polyps (Goodspeed et al., 1987; Pellegrino et al., 1987; Graham et al., 1995; Cullen and Leopold, 1999; Rombaux et al., 2016; Doty, 2019). In addition, anosmia and dysgeusia also present in cases of head trauma and as rare side effects of common medications such as angiotensin-converting enzyme (ACE) inhibitors, angiotensin receptor blockers, dihydropyridine calcium channel blockers, diuretics, and intranasal zinc (Malaty and Malaty, 2013; Schofield and Doty, 2019). Therefore, there is a need to consider the medical history while evaluating individuals with anosmia and dysguesis for the potential suspicion of COVID-19 infection.

To serve as an early diagnostic symptom of infection, it is vitally important to understand the pathophysiology of COVID-19 induced anosmia and dysgeusia. Unlike other etiologies of acquired anosmia, congestion is rare, and onset is rapid in COVID-19. Thus, anosmia may be caused by direct infection and disruption of the olfactory nerve by COVID-19. However, recent data have shown that angiotensin-converting enzyme (ACE2), COVID-19 receptor on host cells, and TMPRSS2, a key protease implicated in viral infection, are not appreciably found in mature olfactory sensory neurons (Figure 1A, Brann et al., 2020). Instead, these receptors are expressed on sustentacular cells, which support olfactory neurons, and at lower levels in olfactory stem cells (Brann et al., 2020). Therefore, it is now speculated that infection of support cells and regenerative stem cells may lead to anosmia rather than by direct effects on the olfactory nerve itself. Loss of supporting cells surrounding olfactory neurons inhibits their function. Moreover, in murine models, juveniles express lower levels of ACE2 and TMPRSS2 compared to adults, consistent with the variable disease severity seen in humans (Brann et al., 2020). Furthermore, coronaviruses are known to infect the central nervous system (CNS) through the cribriform plate, inducing an inflammatory response that can centrally decrease the sense of smell. Once in the CNS, viral spread may induce inflammation in the brainstem, leading to progressive dyspnea and eventually, respiratory failure in severe cases. Indeed, autopsy results have confirmed coronaviral DNA in brain tissue samples (Baig et al., 2020).

**Figure 1:**
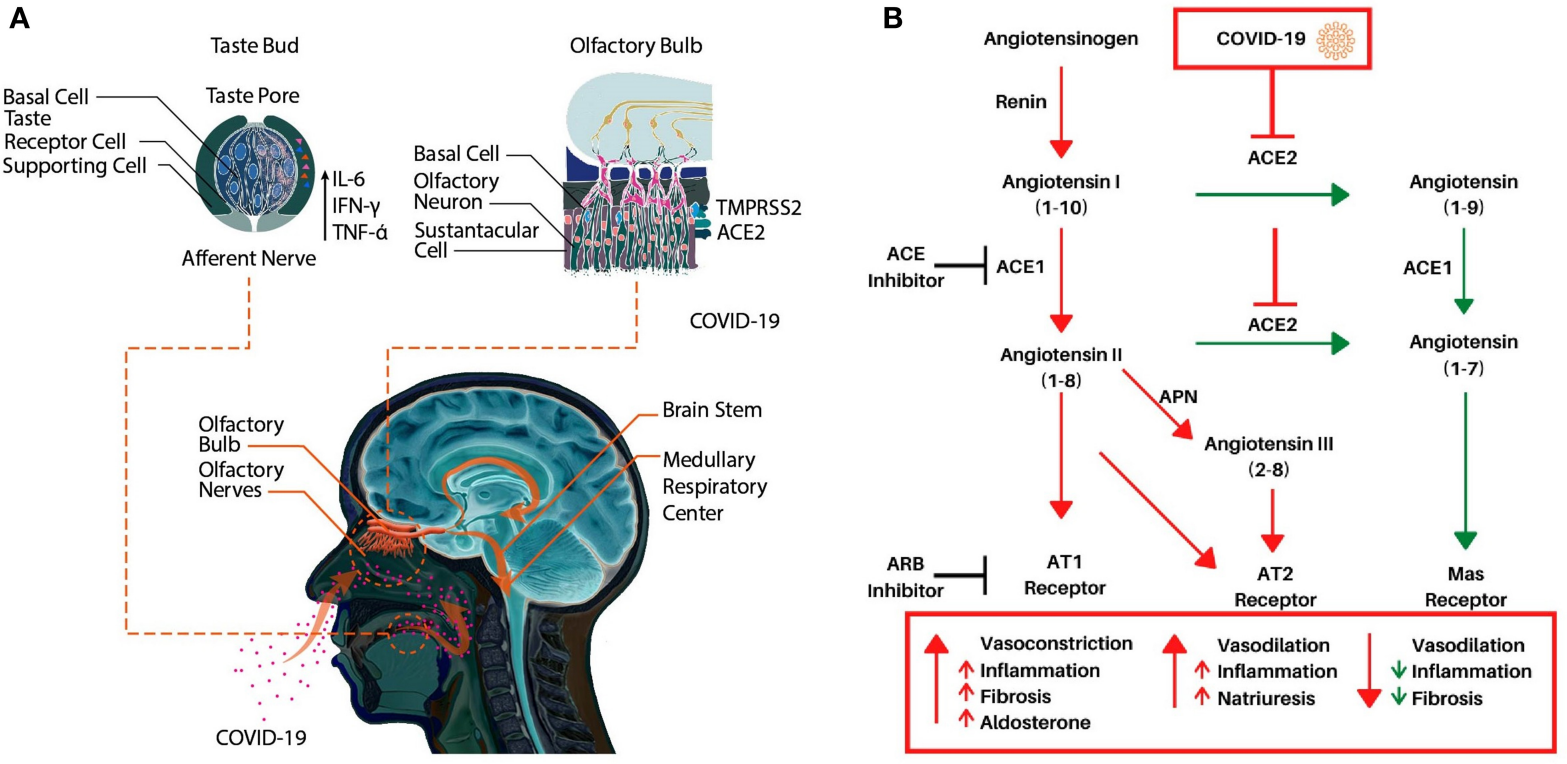
**(A)** A schematic representation of potential molecular mechanisms underlying COVID-19 induced anosmia and dysgeusia. **(B)** The Renin-Angiotensin System (RAS) and its interaction with COVID-19. Renin cleaves angiotensinogen to angiotensin I, an inactive peptide. ACE1 then cleaves angiotensin I to angiotensin II. Angiotensin II can then bind AT1 or AT2 which induces vasoconstriction (AT1), inflammation, and fibrosis. Alternatively, ACE2 cleaves angiotensin I or angiotensin II to angiotensin (1–9) or angiotensin (1–7) respectively, angiotensin (1–9) is then converted to angiotensin (1–7) via ACE1 which then induces vasodilation while decreasing inflammation and fibrosis via the Mas receptor. Angiotensin II can also be converted to angiotensin III via APN which also binds the AT2 receptor. Adapted from Alexandre et al. (2020).

As for dysgeusia, mechanisms of pathogenesis in COVID-19 are not yet known. Taste bud cells have an average life span of 10 days and renew continuously from a population of stem cells in the oral epithelium (Feng et al., 2014; Roper and Chaudhari, 2017). Inflammation, especially pro-inflammatory cytokines such as TNF-α, IFN-γ, and IL-6, can impede stem cell proliferation, and may also reduce the lifespan of mature taste bud cells (Figure 1A) (Cohn et al., 2010). Since high levels of TNF-α, IFN-γ, and IL-6 have been observed in serum of laboratory-confirmed cases of COVID-19 patients, it is reasonable to speculate that these pro-inflammatory cytokines may lead to dysgeusia (Gong et al., 2020). Since the severity of COVID-19 infection is proportional to levels of these pro-inflammatory cytokines, the quality and severity of dysgeusia may help in identifying mild, moderate, and severe cases that can be confirmed using molecular testing.

There is also a possibility that stem and other cells of taste buds express COVID-19 receptors leading to direct infection, cell death, and gustatory dysfunction (Xu et al., 2020). Studies have demonstrated that ACE2 is highly expressed on the oral mucosa and epithelial cells of the tongue, there is a preliminary evidence of ACE2 expression on the apical membrane of taste cells as well (Xu et al., 2020). Importantly, ACE2 mediates the conversion of angiotensin 2 [also known as angiotensin-(1–8)], a pro-inflammatory vasoconstrictor, to angiotensin (1-7), an anti-inflammatory vasodilator (Dandona et al., 2007; Bae et al., 2017; Alexandre et al., 2020). The interaction of COVID-19 with ACE2 downregulates ACE2 expression, which results in a corresponding increase in inflammatory angiotensin II (1–8) via unopposed ACE, and a decrease in anti-inflammatory angiotensin (1–7) due to downregulated ACE2 (Figure 1B) (Silhol et al., 2020). ACE2 also converts angiotensin I [also known as angiotensin-(1–10)] into angiotensin-(1–9), which is then converted to angiotensin-(1–7) by ACE1 (Alexandre et al., 2020). This imbalance between angiotensin II and angiotensin (1–7) is hypothesized to play an essential part in the unfavorable progression of patients with COVID-19 as it may increase inflammation and vasodilation in tissues expressing ACE2 such as the lungs. This imbalance in inflammation may also include the nasal passages, tongue, and oral cavity as these tissues are increasingly known to express ACE2 as well. This further supports COVID-19's direct role in inducing dysgeusia and anosmia, via increased inflammation, cell death, or altering the activity of the RAS system, which is directly implicated in taste conduction (Shigemura et al., 2019).

For clinicians to consider anosmia and dysgeusia as clues for potential identification of COVID-19 positive cases, there is a need for objective measurements to validate and quantify this association. Moreover, patients are generally unable to accurately evaluate their sense of taste and smell, making the standardized exams necessary. Several standardized olfactory function tests such as the Connecticut Chemosensory Clinical Research Center (CCCRC) test and the University of Pennsylvania Smell Identification Test (UPSIT) have been reported in the literature (Doty, 2015, 2019; Baudracco et al., 2020; Carvalho et al., 2020; Garcia et al., 2020; Gurushekar et al., 2020). Briefly, the CCCRC test consists of an odor threshold component and an odor identification component, which are combined for a composite score. For the threshold test, one nostril is closed, and the subject is presented with a solution of dilute butanol or water. The subject must identify which solution contains something other than water in four consecutive trials; if they guess incorrectly, a higher concentration of butanol is then used. The identification test consists of seven scents—baby powder, peanut butter, cinnamon, chocolate, mothballs, coffee, and soap—which test olfactory identification and three stimuli (ammonia, Vicks VapoRub, and wintergreen) which test trigeminal nerve nasal function. However, these are not included in the olfactory function score. The number of correct identifications determines the identification score (Kobayashi et al., 2007). The UPSIT test requires the identification of 40 different smells released via scratching a microencapsulated smell booklet using a pencil (Doty, 2015). Participants then must choose from four possible answers, only one of which is correct. There is another test for clinicians with less experience administering olfactory function tests or for multi-cultural populations, the Odor Stick Identification Test for Japanese (OSIT-J) utilizes easy to use disposable smelling sticks, is faster (~8 min per test), and is demonstrated to work well-cross-culturally (Kobayashi et al., 2007).

It is important to remember that for most people, loss of smell such as during a common cold, may often be perceived as a loss of taste. Thus, objective measurements of dysgeusia in COVID-19 patients are urgently needed to validate and quantify this association.Gustatory function can be evaluated using a validated test where patients rate taste intensity of standardized salty, sweet, sour, and bitter solutions on a scorecard (Landis et al., 2009; Doty, 2018; Vaira et al., 2020). Each of these objective tests has its own scoring method that can be compared to normative populations so that the initial symptoms can be evaluated and then tracked over time. Notably, the gustatory test and the CCCRC have been used to evaluate anosmia and dysgeusia in patients with COVID-19 (Vaira et al., 2020).

Although a possible score based on anosmia and dysgeusia are not intended to replace molecular detection of the virus to manage confinement of patients testing negatives in swab material assessment. However, the presence of anosmia and dysgeusia may help to alert the physicians to repeat the molecular testing if concern for false negative, and the patient to be more prudent to increase the social distance as well as the use of the mask.

In summary, there is a need to identify underappreciated symptoms of COVID-19, especially during the “asymptomatic” stage, and understand the underlying molecular mechanisms that will help in taking preventive measures and facilitate in developing effective treatment modalities. Action now would be especially prescient given the predicted second peak and cyclical nature of COVID-19. There is a need to develop validated scoring tests for more objective evaluations and measurements of anosmia and dysgeusia. In addition, there is a need to explore the role of sex in COVID-19 induced anosmia and dysgeusia in the future investigations. Future critically controlled studies using large cohorts will help in determining whether anosmia and dysgeusia may serve as valuable, early indicators of COVID-19 that may be useful in triaging confirmatory testing or implementing enhanced social distancing and mask-wearing to prevent further spread in the community.

## Author Contributions

All authors listed have made a substantial, direct and intellectual contribution to the work, and approved it for publication.

## Conflict of Interest

The authors declare that the research was conducted in the absence of any commercial or financial relationships that could be construed as a potential conflict of interest.
